# Identification of apolipoprotein B–reactive CDR3 motifs allows tracking of atherosclerosis-related memory CD4^+^T cells in multiple donors

**DOI:** 10.3389/fimmu.2024.1302031

**Published:** 2024-03-20

**Authors:** Payel Roy, Sujit Silas Armstrong Suthahar, Jeffrey Makings, Klaus Ley

**Affiliations:** ^1^ Center for Autoimmunity and Inflammation, La Jolla Institute for Immunology, La Jolla, CA, United States; ^2^ Immunology Center of Georgia, Augusta University, Augusta, GA, United States

**Keywords:** atherosclerosis, antigen-specific, TCR-sequencing, APOB-reactive, CDR3 motifs

## Abstract

**Introduction:**

Atherosclerosis is a major pathological condition that underlies many cardiovascular diseases (CVDs). Its etiology involves breach of tolerance to self, leading to clonal expansion of autoreactive apolipoprotein B (APOB)–reactive CD4^+^T cells that correlates with clinical CVD. The T-cell receptor (TCR) sequences that mediate activation of APOB-specific CD4^+^T cells are unknown.

**Methods:**

In a previous study, we had profiled the hypervariable complementarity determining region 3 (CDR3) of CD4^+^T cells that respond to six immunodominant APOB epitopes in most donors. Here, we comprehensively analyze this dataset of 149,065 APOB-reactive and 199,211 non-reactive control CDR3s from six human leukocyte antigen–typed donors.

**Results:**

We identified 672 highly expanded (frequency threshold > 1.39E-03) clones that were significantly enriched in the APOB-reactive group as compared to the controls (log_10_ odds ratio ≥1, Fisher’s test *p* < 0.01). Analysis of 114,755 naïve, 91,001 central memory (TCM) and 29,839 effector memory (TEM) CDR3 sequences from the same donors revealed that APOB+ clones can be traced to the complex repertoire of unenriched blood T cells. The fraction of APOB+ clones that overlapped with memory CDR3s ranged from 2.2% to 46% (average 16.4%). This was significantly higher than their overlap with the naïve pool, which ranged from 0.7% to 2% (average 1.36%). CDR3 motif analysis with the machine learning–based *in-silico* tool, GLIPHs (grouping of lymphocyte interactions by paratope hotspots), identified 532 APOB+ motifs. Analysis of naïve and memory CDR3 sequences with GLIPH revealed that ~40% (209 of 532) of these APOB+ motifs were enriched in the memory pool. Network analysis with Cytoscape revealed extensive sharing of the memory-affiliated APOB+ motifs across multiple donors. We identified six motifs that were present in TCM and TEM CDR3 sequences from >80% of the donors and were highly enriched in the APOB-reactive TCR repertoire.

**Discussion:**

The identified APOB-reactive expanded CD4^+^T cell clones and conserved motifs can be used to annotate and track human atherosclerosis-related autoreactive CD4^+^T cells and measure their clonal expansion.

## Introduction

Atherosclerosis is the most common pathological condition that underlies many cardiovascular diseases (CVDs), including stroke and myocardial infarction (MI) (1). Atherogenesis involves a gradual accumulation of cholesterol-rich low-density lipoproteins (LDLs) into the subendothelial space of large- and medium-sized arteries. This triggers a cascade of chronic, maladaptive immune cell-mediated inflammatory response that shapes disease progression over decades (2). Recent research has shown that atherosclerosis is accompanied by a breakdown of T-cell tolerance to self (3, 4). Expanded oligoclonal T cells, expressing memory, activation, and effector markers are detectable in human blood and atherosclerotic plaques (5–7). Some of these expanded clones recognize epitopes in apolipoprotein B (APOB) (8, 9). Single-cell transcriptomic analysis of matched blood and plaque samples from the same donors detected effector T cells with signatures of recent activation in both compartments, suggesting systemic recirculation of atherogenic antigen-specific T cells in human atherosclerosis (3).

CD4^+^T cells are the central regulators of humoral and cellular adaptive immunity (10). Activation, expansion, and memory formation of antigen-specific CD4^+^T cells require engagement of unique T-cell receptors (TCRs) by specific epitopes bound to cognate human leukocyte antigen (HLA) Class II molecules (11–13). Due to this strict epitope-receptor specificity, antigen-reactive TCRs serve as unique molecular identifiers that reflect the highly context-dependent immune status of an individual (14). Much of the diversity of human αβTCRs is attributed to the complementarity determining regions (CDRs) (15). Of these, CDR3 spans both the germline encoded variable (V), diversity (D), and joining (J) genes as well as the non-templated insertions or deletions at VDJ junctions (13, 15). This hypervariable region contacts the epitope-HLA complex and thereby encodes critical information about the antigenic specificity of the TCR. Advanced TCR-sequencing pipelines and *in-silico* analysis tools allow precise interrogation of CDR3s from antigen-experienced T cells that are relevant in a specific disease context (16–20).

It is becoming increasingly clear that the disease etiology of atherosclerosis is associated with an autoimmune network of T cells (3, 4, 8). To track these atherogenic cells in humans, it is important to delineate the identities of the epitopes and the TCRs that mediate atherosclerosis-related autoreactivity in humans. We recently reported the identification of six T-cell epitopes in human APOB (9), the core protein component of LDL, and a well-studied atherosclerosis antigen (21). These peptides (APOB_6_) exhibited broad HLA-II–binding preferences and activated proinflammatory and effector/memory CD4^+^T cells in a majority of donors (9). Induction of APOB_6_-specific responses correlated with known CVD risks and with the severity of coronary artery disease (CAD), highlighting that human atherosclerosis is associated with exacerbated responses to these epitopes (9). We had also optimized an *in-vitro* stimulation and flow cytometry–based protocol to enrich and isolate APOB_6_-reactive CD4^+^T cells from human peripheral blood mononuclear cells (PBMCs) and had subjected them to TCR sequencing (9). These sequences and only the top 10 APOB-reactive clones from six donors were previously reported (9).

In this study, we report a more comprehensive and systematic analysis of our published (9) dataset of ~600,000 TCR sequences that were generated from clonally expanded APOB_6_-reactive CD4^+^T cells, non-reactive control cells, and naïve and memory CD4^+^T cells using the immunoSEQ assay from Adaptive Biotechnologies (9). Our analysis helped to define a set of 672 APOB_6_-specific TCRs that exhibited high and specific clonal expansion in the APOB-reactive group but not in the control APOB non-reactive group. Additionally, we conducted CDR3 motif analysis with GLIPHs (22, 23) (grouping of lymphocyte interactions by paratope hotspots) and identified conserved amino acid sequences that were enriched in the epitope-contact sites of APOB_6_-reactive CDR3s. APOB-specific CDR3 clones and motifs mapped to oligoclonal memory CD4^+^T cells from these donors. Some of these APOB-reactive motifs that were enriched in the memory repertoire were also shared across multiple donors. Thus, our dataset allowed assignment of APOB reactivity to TCR sequences within complex repertoire of T cells, enabling tracking and annotation of atherosclerosis-related clones in the general population.

## Results

### Activation-induced marker assay for sorting and sequencing of APOB-reactive CD4^+^T cells from human blood

**Figure 1:**
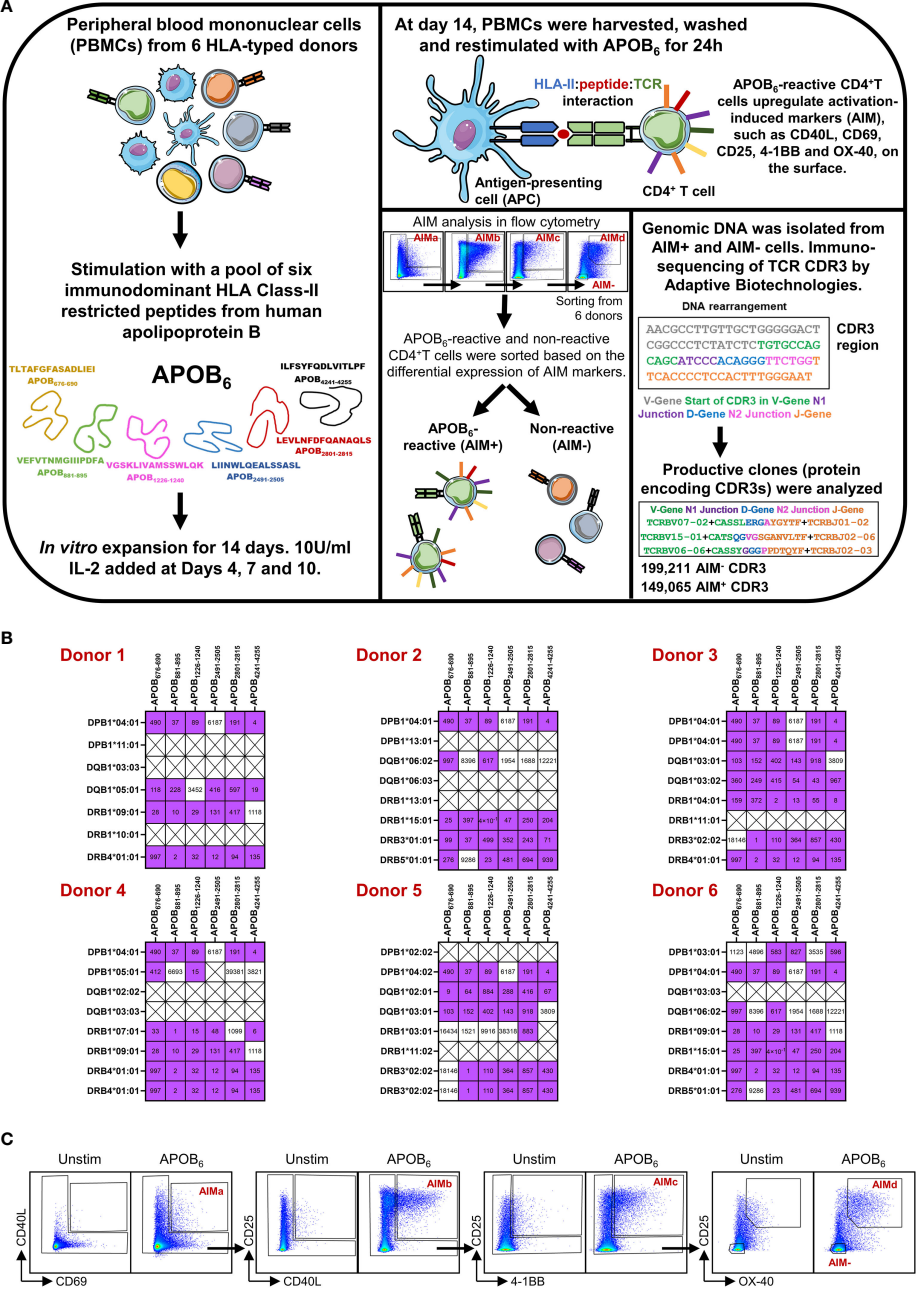
Experimental design and AIM assay. **(A)** Schematic diagram showing epitope-driven clonal expansion of APOB-reactive CD4^+^T cells from PBMCs of six donors, isolation of APOB-reactive, and non-reactive control T cells using the activation-induced marker (AIM) assay, DNA-based sequencing of TCR CDR3 region by Adaptive Biotechnologies and identification of CDR3 rearrangements that are in the correct frame for productive translation to proteins. **(B)** Heatmaps showing binding of the six dominant APOB peptides (columns) to the HLA class II alleles (rows) in each of the six donors. Analysis is based on donor HLA-typing data and experimentally obtained binding profiles of the APOB epitopes. Purple and white boxes denote binding and no binding events, respectively. Binding affinities, nM are labeled in black. Crossed boxes (X) represent conditions when experimental binding data are not available for the specific epitope and donor HLA combination. **(C)** Representative flow cytometry plots showing APOB_6_-induced expression of CD40L^+^CD69^+^ (AIMa), CD25^+^CD40L^+^ (AIMb), CD25^+^ 4-1BB^+^ (AIMc), and CD25^+^OX40^+^ (AIMd) markers on CD4^+^T wells. Unstim (no stimulation) cells were used as negative controls to set the gates. AIM^+^ cells, expressing at least one AIM combination, were considered APOB reactive. AIM^−^ cells, negative for all combinations, were APOB non-reactive.

We briefly describe here the workflow that was used to generate the TCR dataset in our published study (9). To identify epitope-specific CD4^+^T cell clones that are relevant in human atherosclerotic disease (workflow in **Figure 1A**), we examined the CDR3 clones against T-cell epitopes from human APOB protein, one of the few identified atherosclerosis-related autoantigens (21). We focused on six immunodominant APOB epitopes (**Figure 1A**, APOB_676-690_ TLTAFGFASADLIEI, APOB_881-895_ VEFVTNMGIIIPDFA, APOB_1226-1240_ VGSKLIVAMSSWLQK, APOB_2491-2505_ LIINWLQEALSSASL, APOB_2801-2815_ LEVLNFDFQANAQLS, and APOB_4241-4255_ ILFSYFQDLVITLPF), as they were identified as the major antigenic targets of autoreactive human APOB-specific CD4^+^T cells (9). These peptides are broad HLA-II binders (9) and, therefore, were suitable for interrogating the APOB-specific CD4^+^T cell responses in donors expressing diverse HLA-II alleles. We sequenced the HLA locus of the six donors used for TCR repertoire analysis (9). We had previously examined binding of APOB epitopes to an array of common HLA-II alleles (9) using a competition binding assay (24). The threshold for binding was defined as IC_50_<1000 nM (9). Based on these data, we examined the HLA-II–binding information of APOB_6_ to alleles that were specifically expressed by the six donors used for TCR sequencing (**Figure 1B**). This confirmed that each donor expressed four to seven HLA-II alleles (DPB1, DQB1, DRB1, DRB3/4 genes) capable of binding each epitope in our APOB_6_ peptide pool (**Figure 1B**).

To isolate APOB_6_-reactive clones from human blood, our study had described the utilization of an optimized *in-vitro* expansion and restimulation-based protocol (9) that allowed enrichment and selective isolation of rare epitope-specific T cells of interest. We provide here a schematic of the experimental design in **Figure 1A**. As demonstrated in the specificity control experiments (**Supplementary Figure S1**), this assay regime enriches rare autoantigen-specific T cells and improves their detection limit. Our data show that re-stimulation of human PBMCs with APOB peptides elicit robust antigen-specific responses in an IFNγ ELISpot assay (**Supplementary Figure S1A**) only after they have been expanded with the same pool of APOB peptides, but not in control PBMCs that were expanded with IL-2 alone, in the absence of peptides. Similar results were obtained in an intracellular cytokine staining assay (**Supplementary Figure S1B**) in which APOB-induced production of TNF and IFNγ in CD4^+^T cells were observed only when the restimulated PBMCs were first expanded in the presence of APOB peptides and not in stimulus-free expansion sets. As reported in our published study (9), we stimulated PBMCs from six HLA-typed donors (two males and four females; age 24–37 years) with a pool of APOB_6_ and expanded them for 14 days. Expanded PBMCs were washed and restimulated with the APOB_6_ pool using an activation-induced marker (AIM) assay wherein epitope-induced upregulation of T-cell activation markers, CD40L, CD69, CD25, 4-1BB, and OX-40, were examined using flow cytometry. CD4^+^T cells that expressed at least one AIM combination, CD40L^+^CD69^+^ (AIMa), CD25^+^CD40L^+^ (AIMb), CD25^+^ 4-1BB^+^ (AIMc), and CD25^+^OX-40^+^ (AIMd), were considered APOB_6_-reactive (AIM^+^) (9). Those that did not express any combination of these activation markers served as APOB non-reactive controls (AIM^−^). All gates were set using unstimulated sets as background (the gating scheme is shown in **Figure 1C**). Genomic DNA isolated from sorted AIM^+^ and AIM^−^ CD4^+^T subsets were sequenced by Adaptive Biotechnologies using their established immunoSEQ Human TCRB Assay (25). We focused our analysis on productive nucleotide sequences in which the VDJ genes and the junctions in the CDR3 region were in the correct frame for protein translation.

This approach had generated an array of 149,065 APOB-reactive (AIM^+^) and 199,211 non-reactive (AIM^−^) CDR3 sequences from six HLA-typed donors (9). Our published work had reported the identification of only the top 10 APOB-reactive clones from each donor. In this study, we focused on a more comprehensive analysis of this dataset (9) to identify clonally expanded human APOB-reactive CDR3 clones.

### Identification of human APOB-reactive expanded TCR clones

Clonotypic frequencies of the TCRβ CDR3 sequences identified from genomic DNA closely correlate with their actual abundances in the sampled population, because most T cells have only one copy of the somatically rearranged TCRβ CDR3 template (25). Thus, detection of two copies of an identical sequence reflects their shared origin from clonotypic T cells.

**Figure 2:**
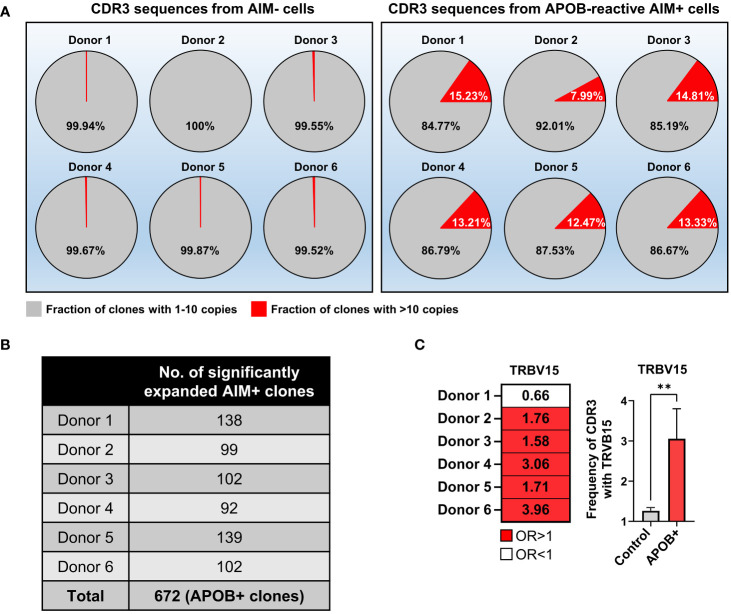
Identification of clonally expanded APOB-reactive TCR clones and their V gene usages. **(A)** Pie charts showing the fraction of productive CDR3 clones with one to 10 copies (gray) or > 10 copies (red) in AIM^−^ (left) and AIM^+^ (right) TCRβ CDR3 sequences from each donor. **(B)** Table showing the number of APOB+ clones from each donor, defined as having >1.39E-03 frequency in the AIM+ subset, significantly (Fisher’s exact test *p* < 0.01) enriched in AIM^+^ compared to the matched AIM^−^ pool in each donor and AIM^+^ versus AIM^−^ log_10_ odds ratio ≥1. Sequences are in **Supplementary Dataset S1**. **(C)** Significant preferential usage of TRBV15 in five of six donors is shown as a heatmap (left) of odds ratios (OR) and bar graphs (right) of TRBV15 gene frequency in APOB+ clones (red) and control clones (gray). Left, red OR > 1, white OR < 1. OR values are labeled in black. Right, mean ± SEM (*n* = 5). Mann-Whitney test, **p < 0.01.

In our array of 149,065 AIM^+^ and 199,211 AIM^−^ productive CDR3 sequences from six donors, we had identified 14,400 unique AIM^+^ clonotypes, 2,492 of which were present at >10 copies (9). A similar copy number–based graded distribution of all unique clonotypes in the AIM^−^ subset (**Supplementary Figure S2**) revealed that the AIM^−^ pools are predominantly enriched in single copy or low copy number (2–10) rearrangements. Clones with >10 copies were ~50 times more frequent in the AIM^+^ compartment (AIM^−^ mean 0.2417%, AIM^+^ mean 12.84%; Mann-Whitney *p* = 0.002). Their frequency ranged between 8% and 16% in the AIM^+^ subsets, as compared to <0.5% in the AIM^−^ control set from each donor (**Figure 2A**). We further analyzed clonal frequencies of all CDR3s in the APOB-reactive AIM^+^ and control AIM^−^ subsets in each donor. We found that a threshold of >1.39E-03 clonal frequency pruned out all AIM^−^ CDR3 clones in at least one donor (the top expanded AIM^−^ clone in donor 2 had a frequency of 1.387E-03). Seven hundred thirty-eight AIM^+^ and 43 AIM^−^ clones had a frequency above this threshold (details in **Supplementary Dataset S1**). To focus on the identification of APOB-reactive clones that were specifically enriched in the AIM^+^ subset, we compared the frequencies of the 738 AIM^+^ clonotypes in AIM^+^ versus AIM^−^ subsets and selected only those with significantly large enrichment in the AIM^+^ group (AIM^+^ vs. AIM^−^ log_10_odds ≥1, False Discovery Rate (FDR)-adjusted Fisher’s exact test *p* < 0.01). This helped to filter out spuriously expanded bystander clones from the AIM^+^ CDR3 repertoire that may have been present at comparable frequencies in both AIM^−^ and AIM^+^ subsets. These enrichment criteria led to the identification of 672 such APOB_6_-reactive (APOB+) clones (**Figure 2B**, number of APOB+ clones per donor). The sequence details, frequencies and copy numbers in the AIM^+^ subset, log_10_odds, and *p*-values are provided in **Supplementary Dataset S1**.

As parts of the TCR V gene contacts with the HLA-II-epitope complex, antigen-specific T cells from different donors can exhibit specific V gene preferences which reflect their underlying biological similarity (15, 23). We compared V gene usages in APOB+ clones (identified in **Figure 2B**) and control AIM^−^ CDR3s in each donor. While multiple functional V genes (annotated using ImMunoGeneTics database (26)) occurred more frequently in the APOB+ clones than control clones from the same donor, only TRBV15 exhibited statistically significant preferential usage in five of six donors (**Figure 2C**).

Thus, we defined the CDR3 sequences and V gene usage of APOB-reactive human CD4^+^T cell clones that showed large and specific expansion in response to APOB_6_ epitopes.

### Detection of APOB+ clones in unenriched T-cell repertoire

T cells with similar reactivity can be tracked across multiple samples using their TCRβ sequence alone (16). High-throughput profiling of TCR repertoire using immunosequencing technology is highly sensitive and can detect even clones with frequencies of 1 in 10,000–100,000 clones (25). Thus, rare epitope-specific clones can be identified within complex mixtures of bulk T cells using a reference set of TCR sequences with known specificities. In our published study (9), we had shown the feasibility of this approach by tracking only the top APOB-reactive clone in our dataset. Individual analysis of other APOB-reactive clones was not done.

**Figure 3:**
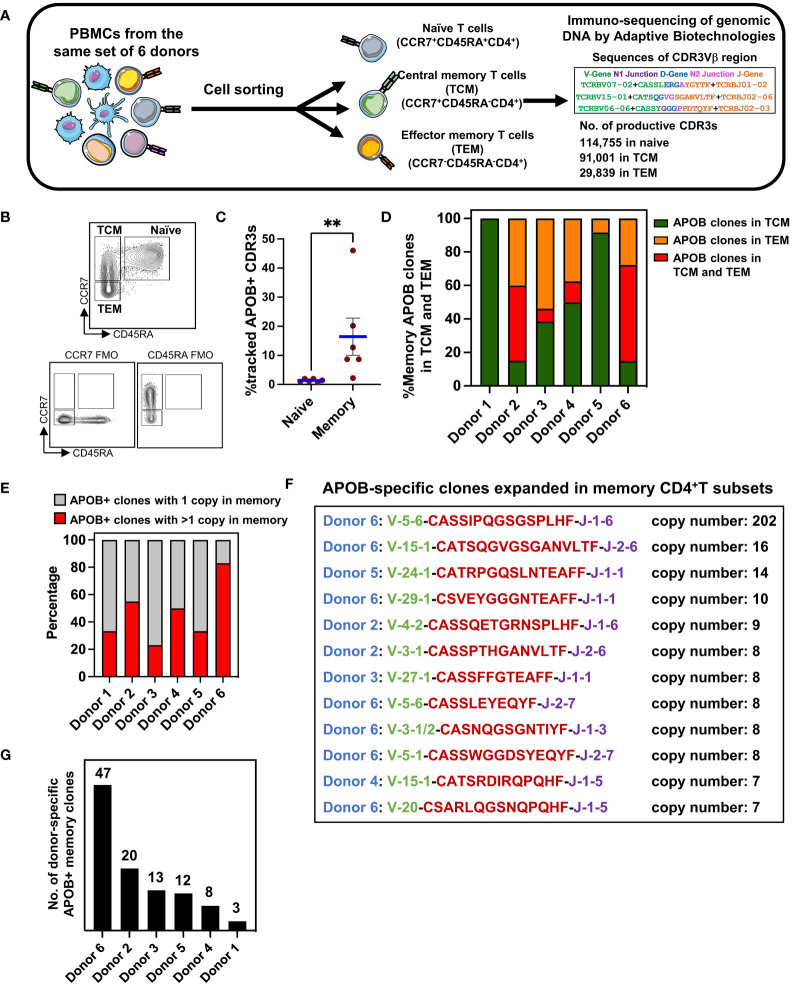
CDR3 sequence-based identification of APOB-reactive clones in unenriched memory TCR repertoire. **(A)** Diagrammatic representation of the workflow for the isolation of human naïve (CCR7^+^CD45RA^+^), central memory (TCM, CCR7^+^CD45RA^−^), and effector memory (TEM, CCR7^−^CD45RA^−^) CD4^+^T cells from blood, DNA-based sequencing, and identification of productive TCRβ CDR3 clones using the immunoSEQ assay from Adaptive Biotechnologies. **(B)** Gating strategy in flow cytometry showing naïve, TCM, and TEM CD4^+^T cells. Gates were set using Fluorescence Minus One (FMO) controls. **(C)** The frequencies of APOB+ clones that were detected in the pool of naïve or memory CDR3s from the same donor. Mean ± SEM are shown. Mann-Whitney test, **p < 0.01.**(D)** Relative frequencies of memory APOB+ clones in TCM (green), TEM (orange) and in both TCM and TEM (red) subsets for each donor. **(E)** Fraction of APOB+ clones with 1 (gray area) or >1 (red area) copy number in the memory compartment of each donor. **(F)** Top APOB+ clones with >6 copies in memory (TCM or TEM). Donor (blue), V gene (green), CDR3 amino acid sequence (red), J gene (purple), and the copy number (black) are shown for each clone. **(G)** Total number of APOB+ memory clones in each donor. Sequence details are in **Supplementary Dataset S2**.

Our defined set of 672 APOB+ CDR3 sequences (**Figure 2B**, **Supplementary Dataset S1**) were identified based on experimentally verified reactivity to six immunodominant APOB epitopes. Based on this, we wished to examine whether this validated set of clonally expanded APOB+ sequences could be tracked in unenriched pools of circulating T cells from the same donors. The generation of naïve and memory TCRs from CD4^+^T cells was described in our published study (9). Briefly, we had obtained PBMCs from the same set of six donors and had stained them with fluorescently labeled antibodies against naïve and memory T-cell markers. Using flow cytometry–based cell sorting, we had isolated CCR7^+^CD45RA^+^ naïve, CCR7^+^CD45RA^−^ central memory (TCM) and CCR7^−^CD45RA^−^ effector memory (TEM) CD4^+^T cells (experimental scheme and gating strategy are shown in **Figures 3A, B**). Genomic DNA from naïve and memory populations were sequenced using the immunoSEQ Human TCRB Assay at Adaptive Biotechnologies (27). We obtained 114,755 naïve and 120,840 memory (91,001 TCM and 29,839 TEM) productive (protein coding) CDR3 nucleotide sequences. These sequences encoded 111,149 naïve; 78,111 TCM; and 18,719 TEM unique clonotypes (9).

While the naïve and memory TCRs were obtained from *ex-vivo* sorted cells, the AIM^+^ and AIM^−^ TCRs were obtained after *in-vitro* expansion and restimulation. During this stimulation regime, the T cells that responded to APOB_6_ and clonally expanded *in vitro* could have originated from a naïve or a TCM or a TEM subset. To test this, we examined the presence of all 672 APOB+ CDR3s within the naïve and memory CDR3 repertoire. Evaluation of APOB+ CDR3 amino acid sequences within naïve and memory CDR3 repertoire of each donor revealed that the frequency of overlap was ~12 times higher for memory CDR3s than for the naïve subset (mean in naïve 1.36, mean in memory 16.4; Mann Whitney *p* = 0.0022) (**Figure 3C**). This confirmed that our dataset of APOB+ sequences could capture the clonal composition of primed CD4^+^T cells that have encountered APOB antigen *in vivo* and were residing in the antigen-experienced memory T-cell compartment. This approach allowed us to annotate individual APOB-reactive clones that represented recall responses in the donors. The size of overlap of APOB+ CDR3s with memory lineage varied from ~2.2% (donor 1) to ~46% (donor 6), suggesting interindividual differences in the clonotypic diversity of the CD4^+^T cells that respond to APOB *in vivo*. The APOB+ clones that were detected in the memory compartment were termed as memory-affiliated APOB+ clones. For each memory-affiliated APOB+ clone, we have provided the sequence details and the clone size in the donor memory pool in **Supplementary Dataset S2**.

Circulating memory T cells reflect the context-dependent heterogeneity of antigen-specific T cells (28, 29). Our cell-sorting protocol combined multiparametric flow cytometry with TCR sequencing, thereby allowing separate analysis of TCM versus TEM clones. Based on this, we analyzed variations in APOB+ sequences among TCM and TEM cells across donors. Most (90%–100%) of the memory-affiliated APOB+ CDR3s mapped to the TCM pool in donors 1 and 5 (**Figure 3D**). In donors 3 and 4, similar proportions of the memory-affiliated APOB+ sequences were found in the TCM and TEM compartment (38.5%–50% in TCM alone and 37.5%–54% in TEM alone, **Figure 3D**). In donors 2 and 6, nearly half (45%–57%) of the memory-affiliated APOB+ clones could be traced to both TCM and TEM CDR3s (**Figure 3D**).

Many of the APOB+ sequences that were traced to the memory pool (**Supplementary Dataset S2**) were expanded (>1 copy number). This varied from 23% in donor 3 to 83% in donor 6 (**Figure 3E**). Sequences of top oligoclonal memory-affiliated APOB+ CDR3s are shown in **Figure 3F**. While most of these clones exhibited moderate expansion in the memory compartment (copy numbers 6–16), >200 copies of one clone were present in donor 6 (**Figure 3F**). This donor also had the highest number of APOB+ clones in the pool of memory CDR3 sequences (**Figure 3G**). All these memory-affiliated APOB+ TCRβ CDR3 sequences (details in **Supplementary Dataset S2**) were private clones and were not shared among the donors.

The overrepresentation of expanded APOB+ clones in the memory compartment and the magnitude (CDR3 copy number) of the APOB-specific memory CD4^+^T cell clones reflect the ongoing APOB reactivity in individual donors.

### Motif analysis of APOB-reactive CDR3s allowed annotation of memory T-cell clones on the basis of shared specificity groups

**Figure 4:**
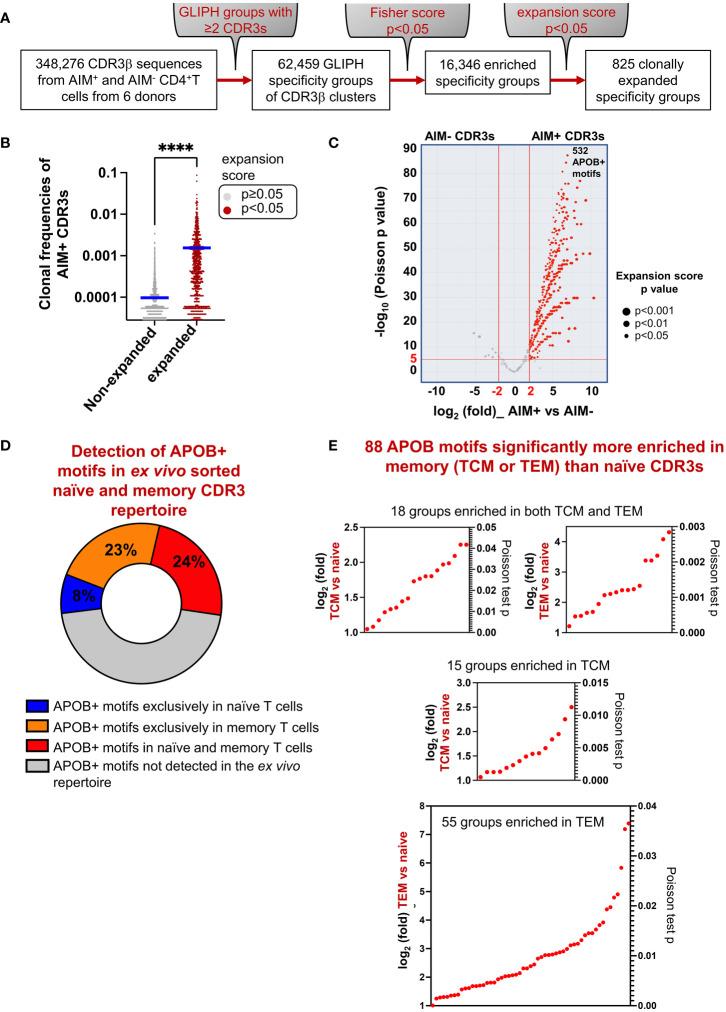
Identification of APOB-reactive CDR3 motifs. **(A)** Flowchart showing motif-based analysis of shared APOB-specificities with the GLIPH 2.0 tool. All 348,276 CDR3 sequences from APOB-specific AIM^+^ and control AIM^−^ CD4^+^T cells were examined. Specificity groups with at least two unique CDR3 members were identified. Statistical significance (Fisher’s exact test *p* < 0.05) of motif enrichment was assessed. Clonally expanded groups with significant expansion scores (*p* < 0.05) were identified. **(B)** Comparison of the frequencies of AIM^+^ CDR3 clones in expanded (brown) versus non-expanded (gray) GLIPH groups that were identified from the expansion scores (expanded *p* < 0.05, non-expanded *p* ≥ 0.05). Mann-Whitney test, ****p < 0.0001.**(C)** Volcano plot showing the relative enrichment of AIM^−^ and AIM^+^ CDR3s in the 825 clonally expanded groups. The *x*-axis represents the log_2_fold differences in the frequencies of AIM^−^ and AIM^+^ clones in each group. The *y*-axis shows the negative log_10_ *p*-values of a Poisson test. Red horizontal and vertical lines indicate the thresholds of |log_2_fold|=2 and −log_10_ *p* ≥ 5 (equivalent to *p* < 1E-05), respectively. Based on this, 532 APOB+ enriched CDR3 motifs (red dots) were identified. Dot size represents expansion score p values from GLIPH. Motif details in **Supplementary Dataset S3**. **(D)** Pie chart showing the relative overlap of the APOB+ motifs in unenriched *ex-vivo* repertoire from bulk CD4^+^T cells. APOB+ motifs, which were not detected in the *ex-vivo* repertoire, are denoted in gray. Details of APOB+ motifs found exclusively in naïve (blue) or memory (orange) CDR3s, or present in both naïve and memory clones (red), are provided in **Supplementary Dataset S4**. **(E)** Scatter plots of log_2_fold (left *y*-axis) and Poisson test *p*-values (right *y*-axis) for 88 APOB+ motifs (red dots on the plots) that were significantly enriched in TCM or TEM clones as compared to the naïve CDR3s. Motif details in **Supplementary Dataset S5**.

Clonotype tracing with exact sequence matching captures only a fraction of all epitope-specific clones. This is because multiple TCRs with non-identical CDR3 sequences have been known to bind the same antigen (30, 31). Analysis of epitope-specific CDR3 arrays in cancer and autoimmune diseases has revealed that TCR clones against the same antigen harbor conserved amino acid motifs at epitope-contact sites (hotspots) in the CDR3 region (18, 22, 32–34). This observation formed the basis for developing the motif analysis tool GLIPH (23), version 2 (GLIPH 2.0) (22). This algorithm scans all input CDR3s and clusters them into specificity groups based on similar amino acid usages at potential epitope-contact sites that were previously annotated from TCR structural data. In this study, we utilized this tool to identify APOB-enriched conserved motifs. GLIPH analysis of all 348,276 CDR3s (149,065 AIM^+^ and 199,211 AIM^−^) in our dataset generated 62,459 groups with at least two distinct CDR3 members (**Figure 4A**). Of these, 16,346 were significantly enriched in our dataset (Fisher’s exact test *p* < 0.05), as compared to a control set of naïve CDR3 sequences, in-built as a reference dataset within GLIPH (23) (**Figure 4A**). An expansion score calculated the statistical significance for any clonal bias in the frequencies of the CDR3 members in a group, as compared to reference. We identified 825 groups with a significant expansion score (*p* < 0.05) (**Figure 4A**). The mean frequency of APOB-specific AIM^+^ CDR3s in the expanded groups was significantly higher than that in the non-expanded group (**Figure 4B**).

To focus specifically on motifs that were enriched in CDR3s with highly significant clonal expansion in the APOB-reactive compartment as compared to non-reactive control, we calculated the relative abundances of AIM^+^ and AIM^−^ CDR3s in each expanded group and calculated their log_2_fold change. The statistical significance of a distribution of AIM^+^ clones with higher frequencies than AIM^−^ controls was determined using a Poisson test. We shortlisted the top 532 groups (**Supplementary Dataset S3**) with AIM^+^ versus AIM^−^ thresholds at |log_2_fold|=2 and -log_10_p=5 (**Figure 4C**). These were annotated as APOB+ motifs.

Next, we tested whether the APOB+ motifs can be traced within unenriched repertoire from bulk CD4^+^T cells. We ran GLIPH with naïve and memory CDR3s from these donors. 7.9% (42 of 532) and 22.7% (121 of 532) of the APOB+ motifs were found exclusively in naïve or memory CDR3s, respectively (**Figure 4D**, **Supplementary Dataset S4**). 23.7% (126 of 532) were found in both naïve and memory groups (**Figure 4D**, **Supplementary Dataset S4**). We compared the relative frequencies of naïve and memory CDR3s in these 126 groups and identified 88 groups (**Figure 4E**, **Supplementary Dataset S5**) with significant expansion in memory CDR3s (|log_2_fold|=1, *p* < 0.05). This comparison was done to select those APOB-reactive motifs that were preferably enriched in the memory pool. Of these, 18 groups had both TCM and TEM members, while 15 and 55 groups were exclusively enriched in TCM or TEM subset, respectively (**Figure 4E**). These and the 121 groups that were exclusively composed of memory CDR3s (**Figure 4E**), together, represented 209 APOB motifs with memory affiliation.

Thus, we defined the identities of APOB-reactive CDR3 motifs. These APOB+ enriched motifs could be traced to the CDR3 array from memory T-cell clones.

### Memory CDR3 sequences from multiple donors share APOB-enriched motifs

**Figure 5:**
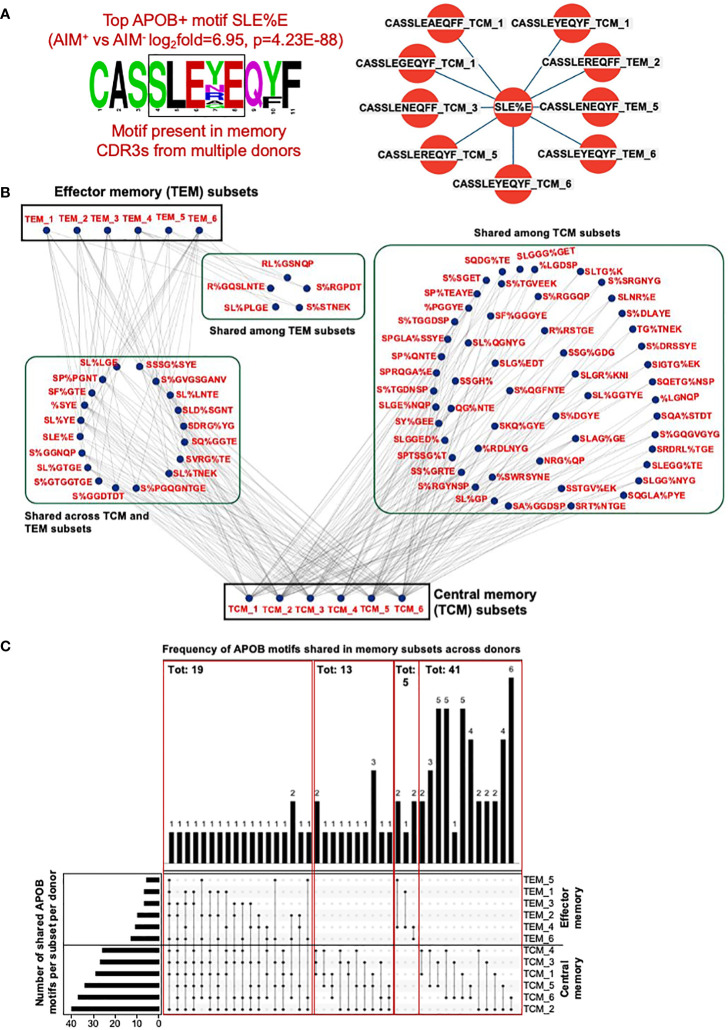
CDR3 clones from memory CD4^+^T subsets share APOB+ GLIPH motifs across donors. **(A)** Left, amino acid sequence logo of all memory CDR3s that contain the APOB+ motif SLE%E (fold change and p value from **Figure 4C**). Color scheme is based on the chemical properties of the amino acids. Polar residues (G, S, T, Y, C, Q, and N) are in green, basic (K, R, and H) in blue, acidic (D, E) in red and hydrophobic (A, V, L, I, P, W, F, and M) residues are in black. Position in the CDR3 sequence in indicated as numbers. Right, sequence, subset (TCM or TEM) and donor (numbers) information for all memory CDR3s with the SLE%E GLIPH motif. **(B)** Cytoscape network analysis of shared APOB+ motifs among TCM and TEM subsets from all six donors. **(C)** Bottom left, total number of shared APOB+ motifs per donor. Bottom center, inter-donor connectivity through sharing of APOB+ motifs is represented as black dots and black joining lines. Bottom right, subset labels to identify connected subsets. Top, frequency of each connection.

Having established that APOB-reactive-enriched CDR3 motifs identified by GLIPH can be found in the memory TCR repertoire, we wanted to examine whether the same motif can be shared by memory TCRs from multiple donors. We first analyzed the top most significantly enriched APOB+ motif SLE%E, where % indicates any amino acid (**Figure 4C**, APOB-AIM^+^ vs. control AIM^−^log_2_fold = 6.95, *p* = 4.23E-88). Memory CDR3 sequences that contained this motif had high-sequence homology (**Figure 5A**, left) and were present in 83% (five of six) donors (**Figure 5A**, right). TCM sequences from donors 1, 3, 5, and 6 and TEM sequences from 2, 5, and 6 harbored the SLE%E motif.

Based on this observation, we analyzed the extent of sharing for all 209 memory-affiliated APOB+ motifs (**Figures 4D, E**). Network analysis and visualization with Cytoscape (35) revealed that many of these groups were present in the memory repertoire from multiple donors (**Figure 5B**). A majority of the sharing was in the TCM pool (**Figure 5B**). Some shared motifs were present in both TCM and TEM subsets, while only five were detected in TEM sequences alone (**Figure 5B**). The numbers of shared APOB+ motifs in the memory compartment varied for each donor (**Figure 5C**, bottom) and was highest for donors 2 and 6 for TCM and TEM groups, respectively (**Figure 5C**, bottom left). Many of the subsets (**Figure 5C**, names in bottom right) were connected through a shared APOB+ motif (**Figure 5C**, dots and lines in bottom center). Of these, 19 connections were among TCM and/or TEM sequences from at least 50% (three of six) donors (**Figure 5C**, top). Thirteen connections came from the sharing of APOB+ motifs across the TCM pool of three donors (**Figure 5C**, top). The remaining groups were shared between two donors, five and 41 for TEM and TCM sequences, respectively (**Figure 5C**, top). Details of all memory TCRs harboring these shared APOB+ motifs (TCRβ CDR3 amino acid sequences, V/J usages, donor and subset-specific frequencies in the memory pool) are provided in **Supplementary Dataset S6**.

While the APOB+ memory CDR3 sequences (**Supplementary Dataset S2**) identified in **Figure 3** were all private clones (no sharing among donors), GLIPH analysis allowed the identification of APOB+ shared motifs that were present in the memory repertoire of multiple donors.

### Identification of top APOB+ public motifs in the memory compartment

**Figure 6:**
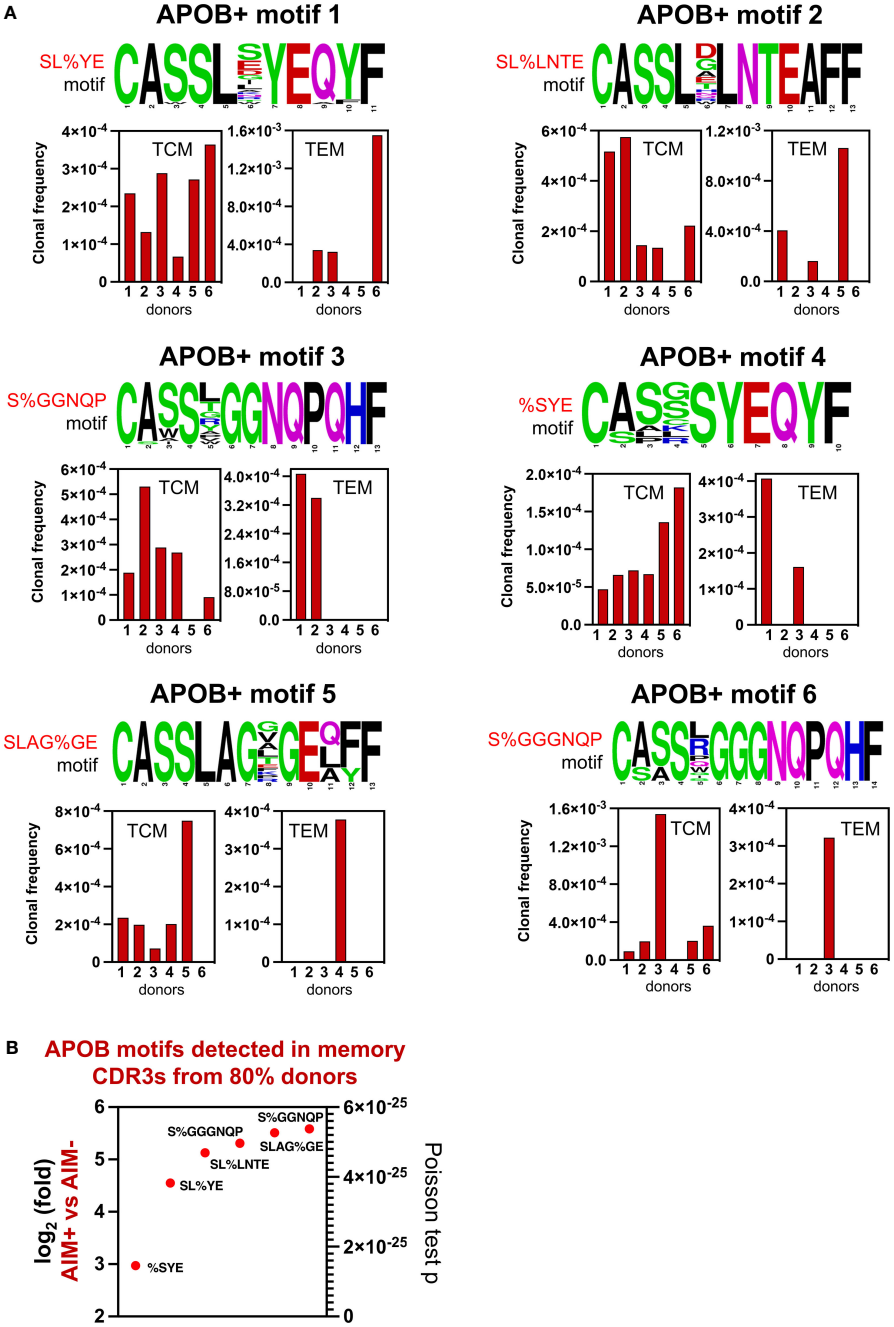
Top 6 APOB+ memory CDR3 GLIPH motifs. **(A)** Top 6 GLIPH motifs showing amino acid sequence logo (top) and donor-specific frequencies of TCM and TEM CDR3s (bottom) that were detected in the memory compartment of at least five donors. Amino acid color scheme as in **Figure 5A**. Position in the CDR3 sequence in indicated below each amino acid. **(B)** Relative expansion in AIM^+^ versus AIM^−^ subset, expressed as log_2_fold (left *y*-axis) and Poisson test *p*-values (right *y*-axis), for these top 6 memory-enriched shared APOB+ GLIPH motifs.

Next, we wanted to identify the APOB+ motifs that were shared across the memory pool of most donors. We analyzed the donor source for all TCM and TEM CDR3s that harbored shared APOB+ motifs (**Figure 5C**, **Supplementary Dataset S6**) and found six of them to be present in >80% of the donors (**Figure 6A**). These APOB+ motifs_1–6_ - SL%YE, SL%LNTE, S%GGNQP, %SYE, SLAG%GE, and S%GGGNQP were detected more frequently in the TCM compartment and were present in at least five of six donors. Sharing of these motifs was also found in the TEM subset, with motifs 1 and 2 being present in the TEM CDR3s of three of six (50%) donors. The motifs 3 and 4 were shared by TEM clones from two donors. Motifs 5 and 6 were restricted to the TEM pool of a single individual. The size of the repertoire, reflected in the clonal frequency of the CDR3s with each motif (**Figure 6A**, bottom panel), varied for different donors. The largest expansions (frequency of ~1.6 in 1,000) were observed for APOB+ motif 1 (SL%YE) in the TEM CDR3s from donor 6 and APOB+ motif 6 (S%GGGNQP) in the TCM pool from donor 3.

Evaluation of the enrichment data from **Figure 4C** revealed highly significant and specific association of these six motifs with APOB-reactive CDR3 clones (**Figure 6B**). Their log_2_fold enrichment in the APOB-specific AIM^+^ compartment, as compared to AIM^−^ controls, ranged from approximately threefold to sixfold, with a statistical significance of at least *p* < 5.6E-25 (**Figure 6B**).

Thus, we were able to identify six APOB-specific CDR3 amino acid motifs that are frequently present in the antigen-experienced memory repertoire and in a majority of the donors, albeit at different proportions.

## Discussion

Atherosclerosis involves chronic and maladaptive inflammation which is associated with a loss of tolerance to self-antigens (2–4). APOB is the most well studied and a clinically relevant atherosclerosis-related autoantigen (21). CD4^+^T cell responses to six immunodominant APOB epitopes correlate with the severity of CAD (9). We had previously generated a dataset of TCR sequences against APOB_6_ epitopes and had reported the top 10 APOB_6_-reactive clones from six donors (9). In this study, we systematically analyzed high and specific clonal expansion of TCR sequences in the APOB-reactive compartment, as compared to the control group. We report the identification of 672 APOB_6_-reactive TCRβ sequences and 532 APOB-enriched CDR3 motifs that allowed the annotation and tracking of clonally expanded APOB-specific CD4^+^T cells in the unenriched memory repertoire of circulating T cells. This is the first study to identify public CDR3 motifs that were shared by APOB-reactive memory TCR sequences in multiple donors.

We analyzed 149,065 TCRβ CDR3 sequences from human CD4^+^T cells that recognized the APOB_6_ epitopes. As control, we examined 199,211 CDR3 sequences from APOB_6_-non-reactive CD4^+^T cells. Both these subsets were isolated by combining multiparametric flow cytometry–based cell sorting techniques with an optimized restimulation-based AIM assay workflow (9). In this assay, stimulation of human PBMCs with APOB_6_ peptide pool induced specific upregulation of T-cell activation markers CD40L, CD69, CD25, OX-40, and 4-1BB on APOB_6_-reactive responding CD4^+^T cells (AIM^+^ subset). APOB_6_ non-reactive CD4^+^T cells did not express these activation markers and were considered as control cells (AIM^−^ subset). We profiled the TCRβ of these sorted cells by sequencing the hypervariable CDR3 using the immunoSEQ assay from Adaptive Biotechnologies (25, 27). Our analyses showed that the frequency of clonally expanded CDR3s was ~50 times higher in the APOB-reactive subset than in the control pool. This high degree of clonal expansion of the APOB-reactive TCRs confirmed that the combination of our AIM assay with immunosequencing techniques can successfully identify antigen-specific oligoclonal T cells. Such a workflow can prove useful to detect rare antigen-specific clones in other disease contexts as well. The detailed workflow and the generation of the TCR dataset was previously reported (9).

To identify the top APOB_6_-reactive clonotypes, we focused our analyses on CDR3 clones that exhibited large (>1.39E-03 clonal frequency in the AIM^+^ subset), specific (AIM^+^ vs. AIM^−^ log_10_odds ≥1), and significant (Fisher’s exact test *p* < 0.01) clonal expansion in response to APOB_6_. This led to the identification of 672 APOB+ clones from six donors. These APOB+ CDR3 sequences did not match other antigen-specific MHCII-restricted CD4^+^ TCRs reported in the publicly available databases VDJdb (36) and McPAS-TCR (37). The details of the sequences that were analyzed from these databases are provided in **Supplementary Dataset S7**. Analysis of V gene preferences revealed a statistically significant preferential usage of TRBV15 in APOB+ clones, as compared to non-reactive control clones.

Next, we tested whether clonally expanded APOB_6_-reactive CDR3 clones can be traced within the complex repertoire of human blood T cells using the TCR sequence as a unique molecular identifier. We analyzed the presence of the 672 APOB+ TCR clones in an array of 114,755 naïve; 91,001 TCM; and 29,839 TEM TCRβ CDR3 sequences from the same donor cohort. We detected a ~12-fold enrichment of APOB+ clonotypes in the memory T-cell compartment, as compared to the naïve subset. This confirmed that the APOB-reactive clones reported in this study map to antigen-experienced CD4^+^T cells and their clonal tracking can inform us about the *in-vivo* status of APOB-reactivity. The frequency of APOB-reactive clonotypes in TCM and TEM compartments and their degrees of clonal expansion varied across the different donors. Thus, TCR sequencing not only allowed us to annotate antigen-experienced APOB-reactive TCRs within the complex repertoire of unenriched bulk CD4^+^T cells, but it also captured the heterogeneity in the magnitude and memory phenotypes of the *in-vivo* CD4^+^T cell response to APOB_6_ epitopes.

Sequence-based tracking of APOB-specific oligoclonal CDR3s was limited because the identified clones were private and not shared among the donors. Previous studies have shown that CDR3 sequences against the same antigen exhibit preferential enrichment of conserved amino acid motifs at specific epitope-TCR contact sites (“hotspots”) (23, 33, 38). These motifs facilitate the identification of antigen-specific TCRs that have similar, but not identical, sequences in the same or different donor. To evaluate whether APOB-reactive TCRs harbored such conserved CDR3 motifs, we analyzed our array of 348,276 APOB_6_-reactive and non-reactive CDR3 sequences with GLIPH2 (22). This well-established motif analysis tool can parse through a vast array of CDR3 sequences and cluster them into specificity groups based on amino acid sequence homology at CDR3 “hotspot” site. We identified 532 amino acid sequence motifs that were specifically and significantly enriched in our dataset of APOB-reactive clonally expanded CDR3s. About 40% of these APOB+ motifs were preferentially detected in the memory T-cell repertoire. Most importantly, multiple donors shared the same APOB+ motif in their memory TCR sequences. Six clonally expanded APOB+ motifs, namely, SL%YE, SL%LNTE, S%GGNQP, %SYE, SLAG%GE, and S%GGGNQP, were present in the memory compartment of >80% of the donors. It is interesting to note that the motif SL%LNTE was also enriched in atherosclerosis-related human cytotoxic CD4^+^T cells reported in another recent study (39) wherein we reported the pathogenic conversion of human regulatory (Treg) cells into inflammatory and cytotoxic exTreg cells under conditions of chronic maladaptive inflammation, as found in CVD. We had reported 345 significantly enriched and expanded CDR3 GLIPH motifs in human exTreg cells (39). Of these, the motif SL%LNTE is an APOB-reactive public motif, as discovered in the present study. This suggests that some human exTreg cells are reactive to immunodominant APOB_6_ epitopes. However, specific reactivity of these motifs to APOB remains to be confirmed experimentally.

In conclusion, we have identified public CDR3 motifs that will facilitate TCR-based tracking of APOB-reactive antigen-experienced CD4^+^T cells in humans. A limitation of this study is that we do not have CVD-related clinical parameters or blood-based laboratory parameters for the donors used in the TCR experiment. Future studies, beyond the scope of this work, will likely reveal more in-depth information about the dynamic correlation between CVD-related clinical parameters and enrichment of APOB+ motifs in atherosclerosis-related pathogenic CD4^+^T cells such as exTregs and effector/memory subsets.

## Materials and methods

### Human samples

Healthy donors, enrolled in the Normal Blood Donation Program, were recruited through the Clinical core at the La Jolla Institute for Immunology (LJI). Written informed consents were obtained from all participants. Financial compensation was provided according to the guidelines approved by LJI’s institutional review board (IRB). Donors were negative for any significant systemic disease or viral infections including hepatitis B or C and HIV. Ethical approval for the study was provided by LJI’s IRB (protocol no. VD-057).

### Preparation of human PBMCs by density-gradient centrifugation

EDTA-coated tubes, containing human blood samples, were centrifuged at 800*g* for 15 min at 24°C with brakes off. The plasma layer on top was removed. An equal amount of serum-free cell culture medium (TexMACS, Miltenyi Biotec, Bergisch Gladbach, Germany) was added and thoroughly mixed. Seven parts of the diluted sample was carefully layered on top of three parts of Ficoll-Paque Plus (MilliporeSigma, Burlington, USA). Tubes were centrifuged at 800*g* for 30 min at 24°C with brakes off. The layer of PBMCs at the interface was transferred into a fresh tube. Cells were washed twice with 1X phosphate buffered saline (PBS; w/o Ca/Mg, Gibco, Billings, USA) by centrifuging at 800*g* for 10 min at 24°C. Cells were counted with a hemocytometer and viability was determined with Trypan Blue. PBMCs were resuspended in CryoStor^®^ CS10 (STEMCELL, Vancouver, Canada) and cryopreserved in liquid nitrogen.

### HLA typing

REPLI-g DNA midi kit (QIAGEN, Hilden, Germany) was used to isolate genomic DNA from human PBMCs using manufacturer’s instructions. For HLA typing, we sent the DNA samples to an ASHI (American Society for Histocompatibility and Immunogenetics)–accredited laboratory at the Institute for Immunology & Infectious Diseases, Murdoch University, Western Australia. We obtained mapped HLA allele information for Class I (A, B, and C) and Class II (DPB1, DQA1, DQB1, DRB1, DRB3, DRB4, and DRB5) genes, as described before (9).

### Peptides

Six 15-mer APOB peptides - TLTAFGFASADLIEI, VEFVTNMGIIIPDFA, VGSKLIVAMSSWLQK, LIINWLQEALSSASL, LEVLNFDFQANAQLS, and ILFSYFQDLVITLPF were synthesized at 2-mg scale (TC Peptide Lab, San Diego, USA) at >95% purity (by reverse-phase high-pressure liquid chromatography). Purity of peptides was confirmed by mass spectrometry.

### Peptide-dependent *in vitro* expansion and restimulation in an AIM assay

Cryopreserved PBMCs from six donors were thawed slowly in a water bath at 37°C. Cells were washed once in PBS (w/o Ca/Mg) by centrifuging at 400*g* for 10 min at 24°C. Cell numbers were determined using a hemocytometer, and viability was examined using the Trypan Blue dye exclusion method. PBMCs were resuspended in TexMACS medium supplemented with 1% penicillin/streptomycin (Thermo Fisher Scientific, Waltham, USA). Cells were plated at a density of 2 × 10^6^ cells/ml in 24-well plates. APOB_6_ peptide pool was added at a dose of 10 μg/ml per peptide and incubated at 37°C with 5% CO_2_. Culture medium was supplemented with 10 U/ml human IL-2 (Invitrogen, Carlsbad, USA) on days 4, 7, and 10. At day 14, cultured cells were harvested and washed with PBS (w/o Ca/Mg). Cells were counted and plated in a flat-bottomed 96-well plate at a density of 1.5 × 10^6^ cells/well. To improve surface detection of CD40L, a CD40 blocking antibody (Novus Biologicals, Centennial, USA) was added at a final concentration of 1 μg/ml. After 15 min of incubation at 37°C with 5% CO_2_, APOB_6_ peptides were added at 10 μg/ml dose for each peptide. No peptides were added to the “unstim” negative control wells. Cells were incubated for 24h at 37°C with 5% CO_2_.

### Flow cytometry and cell sorting

Naïve and memory CD4^+^T cells were isolated from freshly thawed cryopreserved PBMCs. AIM^+^ and AIM^−^ CD4^+^T cells were sorted from cultured PBMCs that were restimulated using the AIM assay (described above). All PBMCs were washed with cold FACS buffer [PBS w/o Ca/Mg, 2% fetal bovine serum (FBS)]. Washed cells were resuspended in a cell-staining master mix containing anti-human (h) Fc-block (1:100, BioLegend, San Diego, USA), BV510 Ghost fixable viability dye (1:1000, Tonbo Biosciences, San Diego, USA), and antibodies against surface markers. Dump channel markers included anti–hCD8a-APC-Cy7 (1:200, clone RPA-T8, BioLegend, San Diego, USA), anti–hCD14-APC-Cy7 (1:200, clone M5E2, BioLegend, San Diego, USA), anti–hCD16-APC-Cy7 (1:200, clone 3G8, BioLegend, San Diego, USA), anti–hCD19-APC-Cy7 (1:200, clone HIB19, BioLegend, San Diego, USA), and anti–hCD56-APC-Cy7 (1:200, clone HCD56, BioLegend, San Diego, USA). T-cell markers included anti–hCD3-PerCp-Cy5.5 (1:200, clone UCHT1, BioLegend, San Diego, USA) and anti–hCD4-Pacific Blue (1:200, clone RPA-T4, BioLegend, San Diego, USA). Memory T-cell markers included anti-hCD45RA-AF700 (1:200, clone HI100, BioLegend, San Diego, USA) and anti–hCCR7-FITC (1:200, clone REA108, Miltenyi Biotec, Bergisch Gladbach, Germany). T-cell activation markers included anti–hCD40L-PE (1:100, clone 24-31, Thermo Fisher Scientific, Waltham, USA), anti–hCD69-BV650 (1:100, clone FN50, BioLegend, San Diego, USA), anti–hCD25-PE-Cy7 (1:100, clone BC96, BioLegend, San Diego, USA), anti–hOX-40-APC (1:100, clone Ber-ACT35, BioLegend, San Diego, USA), and anti–h4-1BB-BV605 (1:100, clone 4B4-1, BioLegend, San Diego, USA). Cells were stained for 60 min at 4°C and then washed with cold FACS buffer (PBS w/o Ca/Mg, 2% FBS). Stained PBMCs were analyzed and sorted using FACSAria II (BD Biosciences, Franklin Lakes, USA) and FACSAria Fusion (BD Biosciences) cell sorters. Voltages were set up using single color-stained cells and compensation beads (UltraComp eBeads™, Invitrogen, Carlsbad, USA). Gates for memory and activation markers were set with Fluorescence Minus One (FMO) and unstimulated controls, respectively. Cells were collected in sort buffer (PBS w/o Ca/Mg, 2% FBS, 0.025M Hepes). Flow cytometry data were analyzed using the FlowJo version 10.8.1.

### TCRβ sequencing from genomic DNA

QIAamp DNA Micro Kit (QIAGEN, Hilden, Germany) was used to extract gDNA from sorted cells using manufacturer’s instructions. DNA samples were shipped to Adaptive Biotechnologies, Seattle, USA for TCRβ sequencing (survey level) using the immunoSEQ platform (27). This process involves a two-step multiplex PCR amplification of CDR3 regions on somatically rearranged human TCRβ chains using optimized set of primers that target the VDJ junctional region in CDR3s. A synthetic repertoire containing all possible V/J templates is used as a built-in control to quantify and correct PCR amplification biases. Processing of raw Illumina sequence reads, filtering, demultiplexing, clustering and mapping of CDR3 sequences, and annotation of VDJ genes using IMGT (ImMunoGeneTics) database were performed by Adaptive Biotechnologies. Final sequence data were made available for download and analysis with their immunoSEQ Analyzer.

### Data analysis

Data analysis and statistical comparisons were done using GraphPad Prism version 9.3.1 and R version 4.0.1. CDR3 nucleotide and amino acid sequences, clonal frequencies, and VDJ gene identities of individual CDR3 rearrangements were analyzed using immunoSEQ Analyzer 3.0. Only productive CDR3 nucleotide sequences, those in the correct frame for protein translation, were considered for downstream analysis. To calculate the odds ratio (OR) for V gene usage, we first counted the number of TCRs with (V+) and without (V−) a particular V gene. For each donor, OR = (no. of V+ in AIM^+^/no. of V+ in AIM^−^)/(no. of V− in AIM^+^/no. of V− in AIM^−^).

CDR3 motif analysis was performed with GLIPH (v.2) (22) using the web tool available at http://50.255.35.37:8080/. GLIPH clusters are local (short stretches of identical residues) or global (not more than 1 aa mismatch) convergence groups in CDR3 amino acid sequences. Enrichment of each GLIPH cluster in our dataset, as compared to a reference set of naïve CDR3s from 12 healthy individuals (22, 23), was evaluated using a Fisher’s exact test. A Fisher’s score of *p* < 0.05 was considered significant. Enrichment of clonal expansion was calculated from CDR3 clonal frequencies by evaluating the statistical significance of the probability that the number of expanded clones in a particular GLIPH group is higher than that in a random sample of an equally sized group from the same dataset. An expansion score of *p* < 0.05 was considered significant. Subset-specific enrichment of motifs was determined by calculating the relative abundance (fold change of clonal frequencies) of members from one subset compared to the other. Statistical significance of the comparison of summed contribution scores from the two subsets was evaluated using the Poisson test.

### Data visualization

Violin plots, scatterplots, pie charts, heatmaps, column, and bar graphs for data visualization were generated using GraphPad Prism version 9.3.1. Volcano plots to visualize differentially enriched CDR3 motifs were generated using R version 4.0.1. UpSet plots to visualize motif sharing across different donor memory subsets were made using tools available at https://www.bioinformatics.com.cn/en, a free online platform for data analysis and visualization. Cytoscape (35), an open-source software platform for network analysis and visualization, was used to generate clusters of homologous TCR members that shared identical CDR3 motifs across different donors and memory subsets. CDR3 amino acid sequence logos were generated with WebLogo (40) version 2.8.2, a free webtool for graphical representation of multiple sequence alignments available at https://weblogo.berkeley.edu/.

## Data availability statement

The datasets presented in this study can be found in online repositories. The names of the repository/repositories and accession number(s) can be found below: GSE261773 (GEO).

## Ethics statement

The studies involving humans were approved by Institutional Review Board at the La Jolla Institute for Immunology. The studies were conducted in accordance with the local legislation and institutional requirements. The participants provided their written informed consent to participate in this study.

## Author contributions

PR: Conceptualization, Data curation, Formal analysis, Investigation, Methodology, Validation, Visualization, Writing – original draft, Writing – review & editing. SA: Conceptualization, Data curation, Formal analysis, Investigation, Methodology, Software, Visualization, Writing – review & editing. JM: Data curation, Investigation, Methodology, Software, Writing – review & editing. KL: Conceptualization, Data curation, Funding acquisition, Resources, Supervision, Writing – review & editing.

## Supplementary material

Supplementary Dataset 1 AIM^+^ and AIM^−^ clones with frequency >1.39E-03 and top APOB+ CDR3 clones after enrichment-based filtering. Donor-specific data on separate tabs.

Supplementary Dataset 2 APOB clones in memory CD4^+^T cells. Donor-specific data on separate tabs.

Supplementary Dataset 3 APOB enriched GLIPH groups. Five hundred and thirty-two GLIPH motifs enriched in APOB-reactive AIM^+^ CDR3 as compared to control non-reactive AIM^−^ sequences.

Supplementary Dataset 4 APOB motifs in CDR3s from bulk CD4^+^T. APOB-enriched GLIPH motifs traced to CDR3 sequences from bulk naïve and memory CD4^+^T cells.

Supplementary Dataset 5 Naive versus memory APOB motifs. Eighty-eight APOB motifs that were significantly enriched in CDR3s from memory compared to naïve CD4^+^T cells.

Supplementary Dataset 6 APOB motifs in memory CDR3 from multiple donors. APOB motifs shared between memory CDR3β sequences from at least two donors.

Supplementary Dataset 7 The details of the CDR3 sequences that were analyzed from the publicly available databases, VDJdb, and McPAS-TCR.
