# Supplementary material for: Identification of apolipoprotein B–reactive CDR3 motifs allows tracking of atherosclerosis-related memory CD4+T cells in multiple donors
Source: Front Immunol. 2024 Mar 20;15:1302031. doi: 10.3389/fimmu.2024.1302031 (PMC10988780; doi:10.3389/fimmu.2024.1302031)
Supplement: Supplementary file 2 [file DataSheet_2.docx]

Supplementary Material

**Identification of apolipoprotein B-reactive CDR3 motifs allow tracking of atherosclerosis-related memory CD4^+^T cells in multiple donors**

Payel Roy^1,2,#^, Sujit Silas Armstrong Suthahar^1,#^, Jeff Makings^1^, Klaus Ley^1,2,*^

^1^La Jolla Institute for Immunology, La Jolla, CA, USA.

^2^Immunology Center of Georgia, Augusta University, Augusta, GA, USA

^#^These authors share first authorship

***Correspondence:**

Klaus Ley, MD

Co-Director, Immunology Center of Georgia (IMMCG)

GRA Bradley Turner Eminent Scholar Chair in Immunology

Professor of Physiology

Medical College of Georgia,

Augusta University,

Immunology Center of Georgia,

1410 Laney Walker Blvd, CN4315,

Augusta, GA 30912.

Email: kley@augusta.edu

Cell: 858-472-7253

Fax: 706-446-0296

## Supplementary Methods

**Human IFNγ ELISpot assay after *in vitro* expansion with IL-2 alone or with IL-2+APOB peptides:** 14-day expanded PBMCs were washed and replated in 96-well ELISpot plates (Millipore) coated with 5 μg/ml mouse anti-human IFNγ (clone 1-D1K) antibody (Mabtech). PBMCs were stimulated with APOB peptides at 10μg/ml. Unstimulated and Phytohemagglutinin-L (1X PHA-L, eBioscience) stimulated sets served as negative and positive controls, respectively. After 24h incubation at 37^0^C, plates were washed six times with PBS containing 0.05% Tween 20 (Millipore Sigma). Plates were then incubated with 1μg/ml mouse anti-human IFNγ (clone 7-B6-1) biotinylated antibody (Mabtech) in PBS containing 0.5% BSA (Millipore Sigma), for 2h at room temperature. Plates were again washed six times with PBS/0.05% Tween 20. Plates were then incubated with VECTASTAIN® Elite ABC-HRP Kit, Peroxidase (Vector Laboratories) in PBS/0.1% Tween 20 for 1h at room temperature. Plates were washed six times with Ultrapure distilled water (Invitrogen). Plates were incubated with 3-amino-9-ethylcarbazole (Millipore Sigma) tablets dissolved in N,N-dimethylformamide (Millipore Sigma), acetate buffer and Hydrogen peroxide solution (Millipore Sigma) for 10 min at room temperature to develop secreted cytokine spots. Plates were dried and wells were imaged using the Zeiss KS ELISpot reader.

**Intracellular cytokine staining (ICS) assay:** After expansion, either with IL-2 alone or with IL-2+APOB peptides, PBMCs were harvested, washed and re-plated in U-bottom 96-well plates. PBMCs were stimulated with APOB peptides at 10μg/ml. Unstimulated sets served as negative controls. After 2h, protein transport inhibitor cocktail (eBioscience) was added at 1X concentration and incubated for an additional period of 4h. After the 6h stimulation period, cells were washed with FACS buffer (PBS w/o Ca/Mg, 2% FBS) and resuspended in staining master mix containing antihuman Fc-Block (Biolegend), fixable viability dye and antibodies against T cell and non-T cell (Dump) surface markers. Viability dye at 1:1000 dilution and antibodies at 1:200 dilutions were used. Cells were stained for 30 min on ice. For intracellular cytokine detection, cells were fixed (eBioscience™ IC Fixation Buffer) for 30min at room temperature. Fixed cells were washed, permeabilized and stained in 1X Perm buffer solution (eBioscience™ Permeabilization Buffer (10X). Cells were stained for TNF (clone Mab11, Biolegend) and IFNγ (clone 4S.B3, Biolegend) cytokines for 45 min at room temperature. Antibodies against intracellular markers were used at a final dilution of 1:50. Single color-stained beads (UltraComp eBeads™, Invitrogen) were used for compensation. Data was acquired on a BD LSR II flow cytometer and analyzed with FlowJo software 10.8.1.

## Supplementary Figures

**Supplementary Figure 1.** **Controls for expansion-based re-stimulation assay protocols**. PBMCs were cultured *in vitro* either in the presence of APOB peptides or without any antigen (indicated as “none” in A or “no stimulus for expansion” in B). 10U/ml IL-2 was added at days 4, 7 and 10. At day 14, PBMCs from both conditions were restimulated with APOB peptides and responses were monitored with an IFNγ ELISpot assay (A) and flow-cytometry-based ICS assay (B). Unstimulated (unstim) cells from both conditions served as negative control in (A) and (B). Additionally, PHA-L was used as a positive control in (A). In (B) the frequencies of TNF and/or IFNγ expressing CD4^+^ T cells in unstimulated sets are shown in blue and the APOB-induced responses in stimulated sets are shown in red.

**Supplementary Figure 2. Copy number-based distribution of AIM^-^ TCR clones.** Total numbers of all unique productive TCR clones identified in AIM^-^ CD4^+^ T cells from individual donors (rows) are shown based on their distribution within a specific range of copy numbers (columns).
